# Assessment of macular function in patients with idiopathic Epiretinal membrane by multifocal Electroretinography: correlation with visual acuity and optical coherence tomography

**DOI:** 10.1186/s12886-017-0621-1

**Published:** 2017-11-28

**Authors:** Meng Gao, Yi Wang, Wu Liu, LiMei Liu, WeiYu Yan, Ju Liu, KeGao Liu, XinXin Liu, YanHua Hu

**Affiliations:** 1 Beijing Tongren Eye Center, Beijing Tongren Hospital, Capital Medical University; Beijing Ophthalmology and Visual Sciences Key Laboratory, Beijing, China; 2 Department of Ophthalmology, Yantai Yuhuangding Hospital, Affiliated Hospital of Medical College, Qingdao University, Yantai, Shandong China; 3 Department of Ophthalmology, Beijing Electric Power Hospital, Beijing, China; 4 Department of Ophthalmology, Kailuan General Hospital, Tangshan, China

**Keywords:** Electroretinography, Epiretinalmembrane, Optical coherence tomography

## Abstract

**Background:**

This study evaluates the macular function changes in patients with idiopathic macular epiretinal membrane (ERM) by multifocal electroretinography (mfERG) and their correlations with visual acuity and central macular thickness (CMT) by optical coherence tomography (OCT).

**Methods:**

Twenty eyes of 20 patients with ERM underwent OCT and mfERG examinations. The response amplitude densities and implicit times of mfERG were compared to the control fellow eyes. Correlation analyses among visual acuity, central macular thickness and mfERG values in the central two concentric rings were performed.

**Results:**

The mfERG P1 response amplitude densities in ring 1–2 and P1 implicit time in ring1 were significantly changed in epiretinal membrane eyes compared with controls (*P < 0.05*). Multivariate stepwise linear regression analyses showed LogMAR visual acuity was significantly correlated with CMT (*P = 0.004*), and also with the P1 amplitude density in ring 1 (*P = 0.002*). CMT showed significant correlation with P1 implicit time in ring 2 (*P = 0.013*).

**Conclusions:**

The mfERG abnormalities show macular function changes and correlate with visual acuity and central macular thickness in eyes with ERM. In first-order mfERG responses, P1 wave changes may be a sensitive functional measurement for ERM patients.

## Background

Idiopathic macular epiretinal membrane (ERM) is a relatively common disorder of the vitreoretinal interface that may occur without clinical signs or cause marked loss of vision and metamorphopsia as a result of covering or distorting the central retina. The functional and anatomical measurements for ERM are usually based on visual acuity and optical coherence tomography (OCT) [1]. Multifocal electroretinography (mfERG),as a noninvasive, objective method to detect regional functional changes in the central retina by measuring electrophysiologic responses, has demonstrated macular function changes in eyes with ERM by several reports [2–6]. To date, however, the value changes in mfERG recordings were inconsistent [3, 4]. The functional measurement characteristic of mfERG for ERM needs further study.

The purpose of the study is to assess first-order mfERG changes in ERM patients and to evaluate the correlations among the visual acuity, OCT parameters and mfERG values.

## Methods

This was an observational study performed between October 2013 and June 2014 at the Beijing Tongren Hospital. Patients with the clinical diagnosis of unilateral idiopathic ERM by ophthalmoscope and OCT were offered enrollment in the study. The patients with secondary ERM, previous history of vitrectomy, other accompanying macular diseases, retinal detachment, retinal vascular diseases, endophthalmitis, or diabetic retinopathy were excluded from this study. The normal fellow eyes without ocular diseases served as controls. Finally, 20 eyes of 20 patients who met the criteria were included for data analysis.

### Ophthalmic examinations

A detailed history, best-corrected visual acuity (BCVA), IOP measurement using noncontact tonometry, slit lamp microscopy and funduscopy were obtained in all participants. BCVA was measured using the Early Treatment Diabetic Retinopathy Study charts at a 4-m distance. Vision results were quantified in logMAR.

### Optical coherence tomography

The scan acquisition protocol for high-definition optical coherence tomography (Cirrus HD-OCT, Carl-Zeiss Meditec, Dublin, CA, USA) was a macular cube 512 × 128 combo across an area of 6 × 6 mm. When the foveal depression was disturbed by the thickened retina, the foveal center was identified as intersection of the point of fixation or the point of maximum outer nuclear layer thickness and minimum inner retinal layer thickness [7]. The central macular thickness (CMT) was defined as the distance between vitreoretinal interface and inner surface of the retinal pigment epithelium.

### Multifocal electroretinography

Multifocal ERG values were recorded with VERIS4.9 software (Electro-Diagnostic Imaging, San Mateo, CA, USA) according to the standard document of the International Society for Clinical Electrophysiology of Vision (ISCEV) [8]. Pupils were fully dilated with 1% tropicamide and 2.5% phenylephrine hydrochloride. The stimulus, consisted of an array of 103 hexagons scaled with eccentricity, was presented on a cathode ray tube monitor with a frame frequency of 75 Hz. The luminance of the stimulus for white was 200 cd/m^2^ and the contrast 99.3%. The bandpass of the filters were 3-100 Hz and amplified with gain of 10^5^. The mfERG responses were recorded with a Burian–Allen bipolar contact lens electrode. Fixation stability was continuously monitored during the testing duration. The first order responses were grouped into 6 eccentric rings. The fovea (ring 1) and parafovea (ring 2) responses of the P1 and N1 waves mainly reflect the macular function and were used for analysis.

### Statistical analysis

Statistical analysis was performed using SPSS software (version 18.0) for Windows (IBM, Armonk, New York, USA). Comparison of data was performed using paired-samplest-test. Pearson correlation analyses were performed for correlation analysis. Multivariate stepwise linear regression analyses by forward selection approaches were performed to investigate the relationship between the visual acuity and other values. A two-sided *P*-value of *<*0.05 was considered statistically significant.

## Results

The mean age of 20 patients with idiopathic ERMs included was 62 years (range: 50–76 years). Two patients were male and 18 female. The mean best-corrected visual acuity (BCVA) of affected eyes was 0.39 ± 0.24 logMAR (range: −0.10 to 0.74 logMAR), which was statistically significant compared with the control fellow eyes (*t = 4.935, P < 0.05*). OCT images showed characteristic pre-retinal highly reflective line beyond fovea in affected eyes. The mean central macular thickness in affected eyes was 470.05 ± 101.91 μm (range: 240-607 μm), while the mean central macular thickness in fellow eyes was 241.15 ± 24.89 μm (range 190-299 μm), the difference was statistically significant (*t = 10.56, P < 0.05*).

### Serial changes in mfERG

**Fig. 1:**
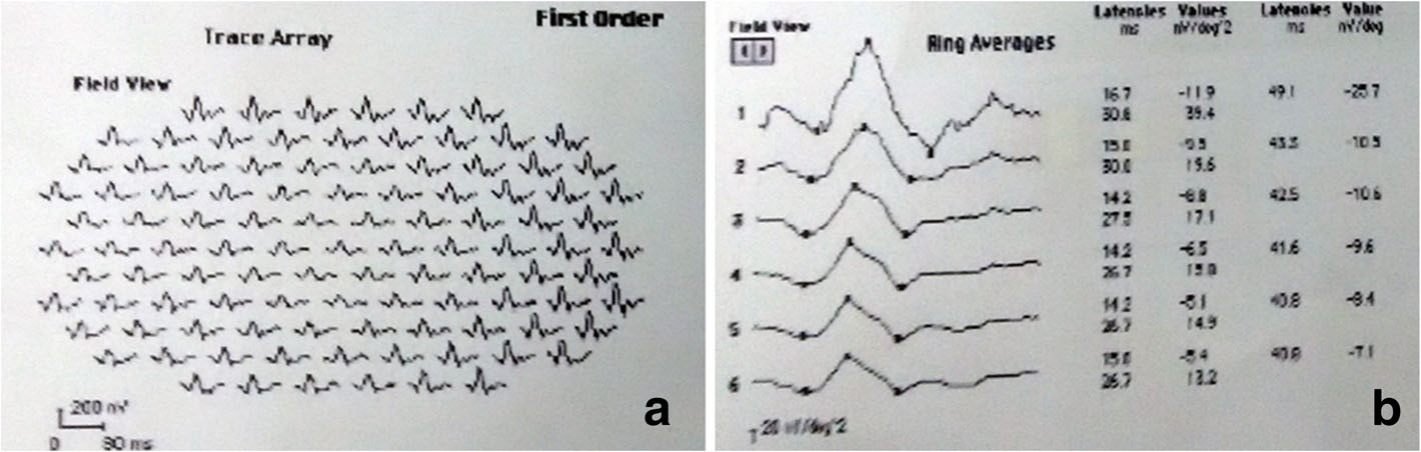
Representative mfERG Images of a patient with idiopathic epiretinal membrane. **a** The first order trace array: the 103 hexagonal elements projected onto the corresponding retina area. **b** Ring averages: The 103 hexagonal elements that compose the six concentric rings. The mfERG responses showed significant reductions in P1 response amplitude densities in ring 1–2 and P1 implicit time in ring1

The changes in mfERG response amplitude densities and implicit times of ring 1 and ring 2 were illustrated in Table 1 and Table 2 respectively. There were significant reductions in P1 response amplitude densities in ring 1–2 and P1 implicit time in ring1 (*P < 0.05*). The representative mfERG images of a case are shown in Fig. 1.

**Table 1:** P1 and N1 amplitude densities in ring 1–2 of ERM patients (mean ± SD, dv/deg^2^)

ring	P1 amplitude density				N1 amplitude density			
	affected	control	t	*p*-value	affected	control	t	*P*-value
1	40.70 ± 15.58	49.87 ± 14.23	3.593	0.002*	−28.06 ± 9.11	−28.75 ± 7.98	−0.284	0.779
2	22.12 ± 7.31	26.04 ± 6.55	3.291	0.004*	−14.23 ± 4.25	−12.07 ± 8.82	1.002	0.329

*P < 0.05 (by paired-samples t-test)

**Table 2:** P1 and N1 implicit times in ring 1–2 of ERM patients (mean ± SD, ms)

ring	P_1_ implicit time				N_1_ implicit time			
	affected	control	t	*p*-value	affected	control	t	*P*-value
1	29.04 ± 1.61	28.13 ± 1.33	−2.244	0.037*	15.77 ± 1.85	14.81 ± 1.63	−1.837	0.082
2	28.35 ± 2.43	27.80 ± 0.99	−0.907	0.376	15.31 ± 1.82	14.59 ± 1.48	1.972	0.063

*P < 0.05 (by paired-samples t-test)

Correlation analysis among LogMAR best-corrected visual acuity, CMT and mfERG values Statistical analysis demonstrated that there was statistically significant correlation between LogMAR BCVA and central macular thickness, P1 amplitude densities,N1amplitude densities, N1 implicit times in ring 1–2 (*r = 0.0614, −0.650, −0.484, 0.543, 0.489, 0.528, 0.582, P = 0.004, 0.002, 0.031, 0.013, 0.029, 0.017, 0.007*; Table 3).

**Table 3:** Correlation analysis among LogMAR best-corrected visual acuity, CMT and mfERG values

LogMAR BCVA correlation factors	eyes	r	*P*-value
CMT	20	0.0614	0.004*
P1 amplitude density in ring 1	20	−0.650	0.002*
P1 amplitude density in ring 2	20	−0.484	0.031*
P1 implicit time in ring 1	20	0.181	0.446
P1 implicit time in ring 2	20	0.318	0.172
N1 amplitude density in ring 1	20	0.543	0.013*
N1 amplitude density in ring 2	20	0.489	0.029*
N1 implicit time in ring 1	20	0.528	0.017*
N1 implicit time in ring 2	20	0.582	0.007*

*P < 0.05 (by Pearson correlation analyses)

The contribution of central macular thickness and mfERG values in defining for LogMAR BCVA was tested through multiple stepwise regression analysis. Parameters have statistically significant correlation with LogMAR BCVA were considered for the model as potential predictors. Multiple linear regression equation: Y = 0.152 + 0.001 X_1_–0.009 X_2_, X_1_ = CMT, X_2_ = P1 amplitude density in ring 1, r^2^ = 0.720.

P1 implicit time in ring 2 and CMT was significantly correlated (*r = 0.545, P < 0.01*), while there were not significant correlation between other mfERG values and CMT (Table 4).

**Table 4:** Correlation analysis between CMT and mfERG values

CMT correlation factors	eyes	r	*P*-value
P1 amplitude density in ring 1	20	−0.11	0.645
P1 amplitude density in ring 2	20	−0.017	0.945
P1 implicit time in ring 1	20	0.205	0.386
P1 implicit time in ring 2	20	0.545	0.013*
N1 amplitude density in ring 1	20	0.148	0.534
N1 amplitude density in ring 2	20	0.121	0.613
N1 implicit time in ring 1	20	0.098	0.68
N1 implicit time in ring 2	20	0.357	0.123

*P < 0.05 (by Pearson correlation analyses)

## Discussion

Previous studies [2, 3, 6] in the use of mfERG in assessing idiopathic ERM have found the mfERG values were reduced in the central retina. Consistent with previous studies, our results demonstrated that mfERGchanges in ring 1 and ring 2. The observed reduction in mfERG values indicates visual function impairment associated with idiopathic ERM occurred in areas beyond fovea, which was consistent with extensive macular edema to perifovea in OCT figures.

Significant changes were found in P1 amplitude densities in ring 1–2 and P1 implicit time in ring1 in eyes with ERM compared to the control fellow eyes, while there were no significant differences in N1 amplitude densities or implicit times. This result is similar to previous report [3, 4]. TheN1 and P1 mfERG wave forms are believed to originate from the outer retinal layer and the inner retinal layer respectively [9, 10]. Previous studies [11, 12] have reported inner retina had the most variability of thickness in eyes with ERM. It seems that P1 wave changes were the main deterioration in mfERG associated with ERM, while the N1 waveforms were not significantly affected.

The retinal thickening and edema in ERM patients lead to vision losses. The visual acuity was negatively correlated with the central macular thickness, which is consistent with previous studies [13]. Multiple stepwise regression analysis showed P1 amplitude density in ring 1 was also correlated with the visual acuity. Tanikawa et al. studied 30 patients with unilateral idiopathic ERM using focal macular ERGs and reported that there was a significant correlation between the b-wave amplitude and the visual acuity [14], while the P1 waveform of the mfERG appear to be generated by the same cells generating the b-wave of the full-field ERG [10]. Although the cellular impairment mechanism underlying ERM remains unclear, we suggested that P1 wave changes in this study may reflect the inner retina layer damages induced by ERM, which seemed to play an important role in vision loss.

The mfERG values might be associated with numerous factors. Previous studies have found that abnormalities in the P1 latency disorders might reflect dysfunctions of the inner retinal layers and Müller cells [15]. We demonstrated significant correlation between P1 implicit time in ring 2 and CMT. In contrast, other studies [3] reported there was no statistically significant correlation between the mfERG values and CMT. This discrepancy may relate to the consistent damage caused by prolonged duration and thickened retinal thickness in this study.

The limitations of our study include a small sample size. This might have limited the power in detecting photoreceptor statues and other influence factors, which may have an impact on ERG values and statistical analysis. The mechanism of mfERG impairment related to ERM may not be straightforward. The multifocal ERG abnormalities as described hereneeds further study.

## Conclusion

In summary, our findings showed mfERG abnormalities appear to demonstrate subtle macular function changes and correlate with visual acuity and central macular thickness in eyes with ERM. In first-order mfERG responses, P1 wave changes may be a sensitive functional measurement for ERM patients.

## Abbreviations

BCVA: Best-corrected visual acuity
CMT: Central macular thickness
ERM: Epiretinal membrane
mfERG: Multifocal electroretinography
OCT: Optical coherence tomography
